# Neurotoxicity after hematopoietic stem cell transplant in multiple sclerosis

**DOI:** 10.1002/acn3.51045

**Published:** 2020-04-18

**Authors:** Simon Thebault, Hyunwoo Lee, Gauruv Bose, Daniel Tessier, Mohammad Abdoli, Marjorie Bowman, Jason Berard, Lisa Walker, Carolina A. Rush, Heather MacLean, Ronald A. Booth, Sridar Narayanan, Douglas L. Arnold, Vincent Tabard‐Cossa, Harold L. Atkins, Amit Bar‐Or, Mark S. Freedman

**Affiliations:** ^1^ The University of Ottawa and The Ottawa Hospital Research Institute Ottawa ON Canada; ^2^ Department of Neurology and Neurosurgery McGill University Montreal QC Canada; ^3^ Department of Physics The University of Ottawa Ottawa ON Canada; ^4^ Department of Neurology and Center for Neuroinflammation and Experimental Therapeutics Perelman School of Medicine, and the Children’s Hospital of Philadelphia University of Pennsylvania Philadelphia Pennsylvania

## Abstract

**Objective:**

Accelerated brain volume loss has been noted following immunoablative autologous hematopoietic stem cell transplantation (IAHSCT) for multiple sclerosis. As with other MS treatments, this is often interpreted as ‘pseudoatrophy’, related to reduced inflammation. Treatment‐related neurotoxicity may be contributory**.** We sought objective evidence of post‐IAHSCT toxicity by quantifying levels of Neurofilament Light Chain (sNfL) and Glial Fibrillary Acidic Protein (sGFAP) before and after treatment as markers of neuroaxonal and glial cell damage.

**Methods:**

Sera were collected from 22 MS patients pre‐ and post‐IAHSCT at 3, 6, 9, and 12 months along with 28 noninflammatory controls. sNfL and sGFAP quantification was performed using the SiMoA single‐molecule assay.

**Results:**

Pre‐IAHSCT levels of sNfL and sGFAP were elevated in MS patients compared with controls (geometric mean sNfL 21.8 vs. 6.4 pg/mL, sGFAP 107.4 vs. 50.7 pg/mL*, P* = 0.0001 for both). Three months after IAHSCT, levels of sNfL and sGFAP increased from baseline by 32.1% and 74.8%, respectively (*P* = 0.0029 and 0.0004). sNfL increases correlated with total busulfan dose (*P* = 0.034), EDSS score worsening at 6 months (*P* = 0.041), and MRI grey matter volume loss at 6 months (*P* = 0.0023). Subsequent NfL levels reduced to less than baseline (12‐month geometric mean 11.3 pg/mL *P* = 0.0001) but were still higher than controls (*P* = 0.0001). sGFAP levels reduced more slowly but at 12 months were approaching baseline levels (130.7 pg/mL).

**Interpretation:**

There is direct evidence of transient CNS toxicity immediately after IAHSCT which may be chemotherapy mediated and contributes to transient increases in MRI atrophy.

## Introduction

Immunoablation and autologous hematopoietic stem cell transplantation (IAHSCT) is an intensive yet efficacious therapy used for particularly aggressive cases of multiple sclerosis (MS).^1^ Specific regimes utilized by different centers vary mainly in both intensity of treatment conditioning and durable efficacy of treatment responses.^2^ The Canadian regimen^3^ is considered particularly intensive and involves high‐dose cyclophosphamide and busulfan as conditioning agents. These drugs are selected not only for their peripheral myeloablative and lymphoablative actions but also for their CNS penetrance and toxicity to microglia involved in MS pathogenesis. However, these are potent agents, with potential for iatrogenic toxicity. We previously investigated the rapid loss of brain volume in the first 6 months following IAHSCT conditioning.^4^ In this study, our group concluded that “pseudoatrophy” (brain volume loss due to the reduction in inflammation) may not fully explain the transient increase in rate of brain volume loss; we hypothesize that CNS toxicity related to the treatment itself may be contributory.^5^

Chemotherapy has been known to be associated with neurotoxicity and cognitive impairment.^6^ The colloquial terms ‘chemo brain’ and ‘chemo fog’ are commonly used by patients to describe subjective disturbances in cognition and memory experienced around the time of treatment,^7^ The effect is dose responsive, with higher doses of chemotherapy related to worse cognitive scores.^8^ Furthermore, the chemotherapeutic agents used for immunoablation in MS are in part selected for their ability to penetrate the CNS and neurotoxicity invitro.^9^ Petzold *et al* ^10^ found that after conditioning with horse antithymocyte globulin and whole body irradiation, a particularly intensive regimen no longer routinely used for MS, blood concentrations of neurofilament heavy chain (NfH) increased in 79% of MS patients and 49% of hematological malignancy patients. In our cohort of 24 Canadian patients who underwent IAHSCT for aggressive MS,^11^ the highest rates of early post‐treatment MRI volume loss were found in patients who received the highest doses of busulfan chemotherapy and also those who had the greatest burden of white matter lesions on T1‐weighted MRI (T1‐weighted lesion) prior to treatment.^4^

Recent advances in sensitive detection methodologies using digital immunoassays have enabled reliable measurement of several protein biomarkers relevant to neurology not previously detectable in blood samples.^12^, ^13^ Levels of serum Neurofilament light chain (sNfL) have attracted the most interest: this neuroaxonal intermediate protein reflects CNS damage in several conditions including traumatic brain injury,^14^ neurodegenerative conditions,^15^ and MS.^16^ Similarly, levels of serum Glial Fibrillary Acidic Protein (sGFAP) is a marker of astrocytic turnover or damage.^17^ Here, we measured levels of sNfL and sGFAP in stored sera from our IAHSCT cohort, hypothesizing that increased levels would correlate with the MRI metrics as well as available clinical indices, and support the notion that the loss of brain volume was due to chemotherapy‐induced neurotoxicity.

## Subjects, Materials, And Methods

### Patients with aggressive MS undergoing IAHSCT

The Canadian BMT in MS study has been previously described in detail.^11^ Briefly, it was a phase II, single‐arm, nonrandomized control study of IAHSCT (*n* = 24) performed at three hospitals in Canada, in patients with aggressive MS who had a poor predicted 10‐year prognosis^18^, ^19^ (ClinicalTrials.gov, NCT01099930). Owing to an adverse event related to busulfan toxicity midway through patient recruitment to the study, there was a protocol change resulting in a reduction in total busulfan dose delivered to subsequent patients. Detailed follow‐up of the initial study included predefined and scheduled clinical, laboratory, and MRI examinations starting 5 months before the IAHSCT and continuing for at least 3 years. The study was then extended for monitoring for more than a decade.

Longitudinal MRI measures (T1‐weighted sequences pre‐ and postgadolinium (Gd), T2‐weighted sequences and brain volume metrics) were obtained at predefined time intervals. Two pretreatment MRI scans were performed with a minimum interval of 2 months, followed by further scans at 1, 2, 4, 6, 9, and 12 months, then every 6 months until 3 years, thereafter every 12 months, to a maximum duration of 10.5 years. Extensive neuropsychological data were available at baseline, and then 6 and 12 months following treatment covering multiple cognitive domains including information processing speed (tested with Symbol Digit Modalities Test and Computerised Test of Information Processing, Paced Auditory Serial Addition Test), visual memory (Brief Visuospatial Memory Test– Revised), verbal memory (Hopkins Verbal Learning Test–Revised), Visual Perception (Judgement of Line of Orientation), executive function (Delis‐Kaplan Sorting Test, Letter fluency), and language (semantic fluency by animal naming). Similarly, we had quantitative fatigue (Fatigue Impact Score) and quality of life (MS‐QOL‐54, physical and mental composites) data at the same time points.

From the 24 patients, serum was available for 21 patients at baseline, 15 at 3 months, 19 at 6 months, 15 at 9 months, and 22 at 12 months post‐IAHSCT. Collected between 2001 and 2012, blood samples had been processed immediately following collection by a dedicated research technologist, aliquoted (to avoid freeze‐thaws) and stored in the same −80°C freezer.

### Noninflammatory controls

We identified 28 patients with noninflammatory illnesses with similar ages the patients undergoing IAHSCT and available serum collected over the same period as the IAHSCT samples (2000–2012). These were collected with appropriate consent and had undergone identical processing and storage at −80 degrees as the IAHSCT samples. Their diagnoses were as follows: migraine (*n* = 7), conversion/somatization (*n* = 6), fibromyalgia (*n* = 5), anxiety (*n* = 4), Bell’s palsy (*n* = 2), labyrinthitis (*n* = 2), and depression (*n* = 2).

### Research questions (Level II evidence)

Primary research question: *In this cohort of patients with aggressive MS treated with IAHSCT, can we detect early post‐treatment evidence of neuroaxonal and glial toxicity as evidenced by rises in sNfL and sGFAP levels?*

Secondary research question: *Do the greatest increases in sNfL and sGFAP correlate with chemotherapeutic dose and the magnitude of early MRI brain volume loss?*

### Quantification of sNfL and sGFAP

sNfL and sGFAP levels were quantified simultaneously in a single batched experiment with samples run in duplicate according to manufacturers’ instructions with appropriate standards and internal controls. Quanterix® commercially available Neurology 4‐Plex A (NfL, Tau, GFAP, and UCHL‐1; catalog number: 102153) was run in the fully automated ultrasensitive SiMoA HD‐1 Analyzer (Quanterix®).

### MRI analyses

#### T1 and T2 lesion volumes

MRI lesion‐based measurements included: number and volume of hypointense T1‐weighted lesions (precontrast), number and volume of gadolinium‐enhancing (Gd) lesions (T1 postcontrast), number of new or enlarging T2‐weighted lesions, and volume of T2‐weighted lesions.

#### MRI brain volume changes (whole brain, grey matter, and white matter)

Longitudinal brain volume changes were calculated in individual patients using pairs of precontrast T1‐weighted images. All measurements were made with respect to the baseline MRI scans. Percentage whole brain volume changes were calculated using FSL‐SIENA package.^20^ Grey and white matter volume changes were separately calculated using Jacobian Integration, which is a nonlinear registration‐based method.^21^

### Statistical analyses

Data analysis was performed using Graphpad ® Prism 8.2 software. For all analyses, a 95% significance level was used, and *P*‐values were two‐tailed. All statistics were overseen by a statistician available through the Ottawa Hospital Methods Centre. To analyze the changes in serum biomarker levels over time, we selected a mixed‐effects model approach over basic repeated measures ANOVA so that we could analyze the data without needing to impute missing values. As this approach is only appropriate for parametric statistical analyses, we first assessed the data for normality and log normality (using the natural base, ln) with the D'Agostino & Pearson test. These overall distributions of sNfL and sGFAP concentrations were non‐normally distributed because of biologically plausible higher values, and natural log‐transformation (ln) produced plausibly normal distributions. Ln transformation of blood biomarker levels is a well‐established technique for blood biomarker data.^22^ We then used a mixed‐effects model to compare the means of all natural log (ln)‐transformed columns using Tukey’s HSD test to assess for significant differences. Average sNfL levels for each group are quoted as the geometric mean (hereafter, geomean). This approach is more statistically rigorous than analyzing the data in a nonparametric fashion using multiple‐paired/unpaired t tests with Wilcoxon/Mann–Whitney tests.

To assess for correlations of baseline serum biomarker levels with MRI, neurophysiologic and demographic variables, Spearman (nonparametric) correlation was selected as this was more appropriate for comparing biomarker levels with other variables. In order to examine the relationship between serum NfL and cognitive domains, baseline to 6‐month differences in both raw performance scores and reliable change index (RCI) scores for each test were correlated with changes in sNfL from baseline to 3, 6, and 12 months. RCI scores allow for the assessment of an individual's change in performance over time, while adjusting for practice effects that can occur as a result of serial cognitive testing.^23^ To conduct binary comparisons of continuous variables (busulfan dose and 3‐month ΔsNfL), the median was selected as a cut‐off; values above the median were considered ‘high’ and below the median were considered ‘low’. This was particularly appropriate in the case of total busulfan doses; as described above, midway through the study there was a protocol change such that the distribution of busulfan doses was binomial. For such comparisons, we used either unpaired t tests (Mann–Whitney) or the mixed‐effects analysis with Geisser‐Greenhouse correction.

### Registrations, patient consents, approvals, and data availability

Ethics and protocols in this study had been approved by Regional Ethics Boards of participating institutions as part of the original study, ClinicalTrials.gov Identifier: NCT01099930.^11^ All participants had given informed written consent.

Individual deidentified participant data from this study will be shared with qualified investigators on written request to the corresponding authors.

## Results

### Baseline characteristics of IAHSCT patients and noninflammatory controls

Baseline characteristics of patients with aggressive MS and controls are outlined in Table 1.

**Table 1:** Baseline demographic and clinical details of patients and controls

	Aggressive MS treated by IAHSCT	Noninflammatory controls
Number of patients	22	28
Mean age at baseline sampling (range)	33.2 (23–44)	35.9 (25–46)
Gender: female, male	13,9	21,7
Mean years disease duration at baseline (range)	7.4 (1–21)	NA
Median EDSS score at baseline (range)	5.0 (2–6.5)	NA
Clinical subtype RR, SP	12, 10	NA

EDSS, Expanded Disability Status Scale; IAHSCT, Autologous Hematopoietic Stem Cell Transplantation; RRMS, Relapsing Remitting MS; SPMS, Secondary Progressive MS.

### Changes in serum biomarkers in the first year following IAHSCT compared with controls

**Figure 1:**
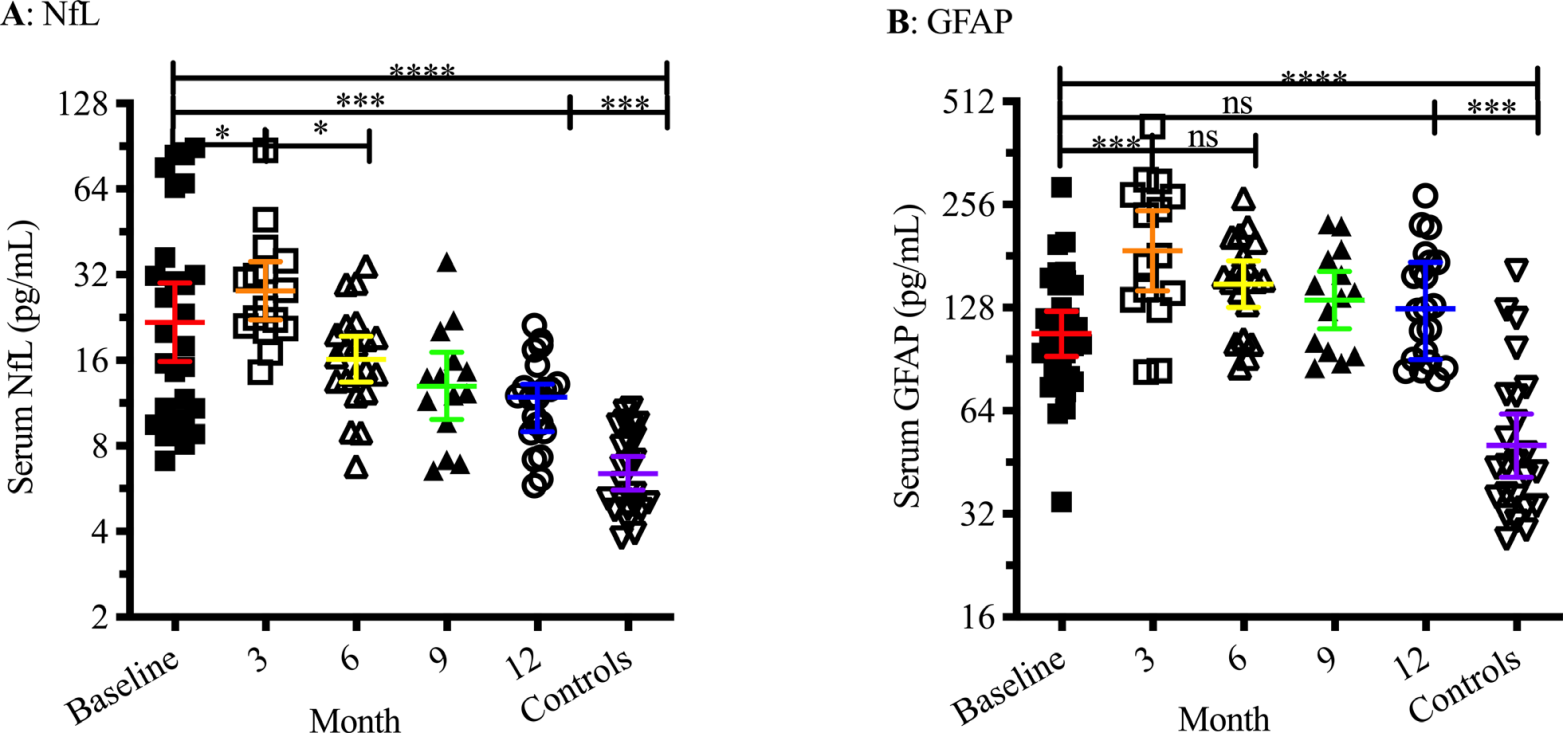
Serum levels of both NfL (A) and GFAP (B) were elevated in patients with aggressive MS prior to IAHSCT compared with controls (*P* = 0.0001). Three months post‐IAHSCT, mean levels of both biomarkers transiently increased relative to pretreatment levels (NfL *P* = 0.029, GFAP *P* = 0.0004). While NfL levels durably went down to less than pretreatment levels by 12 months (*P* = 0.0005), levels of GFAP remained similar to pretreatment levels. Bars and error bars denote geometric mean and 95% confidence interval. ‘ns’ denotes a nonsignificant *P* value. *, **, ***, **** signify a *P*‐value ≤ 0.05, 0.01, 0.001, 0.0001 respectively.

Pre‐IAHSCT levels of sNfL were elevated compared with controls (GeoMean 21.8 vs. 6.4 pg/mL, Unpaired t test *P* = 0.0001, Fig. 1A). Following treatment, levels of sNfL increased relative to baseline in 13/15 patients (geomean 28.8 pg/mL, *P* = 0.029, 95% CI for the ln‐transformed mean difference 0.029–0.7173). The baseline to 3‐month increase in geometric mean NfL from 21.8 to 28.8 pg/mL represented a 32.1% increase. There were two patients with a baseline to 3‐month decrease in levels; both of these individuals had some of the highest NfL levels at baseline, 67 and 89 pg/mL, respectively. At 6 months, geomean sNfL levels had reduced from their 3‐month high to 16.2 pg/mL (*P* = 0.0284, CI −1.033 to −0.03584). By 12 months, the geomean NfL level was 11.26 pg/mL, and was below pretreatment levels in 19/22 patients (*P* = 0.0005, CI −1.090 to −0.2161), but remained elevated relative to controls (Unpaired t test *P* = 0.0001). There were three patients with a baseline to 12‐month increase in levels; all three had relatively low NfL levels to begin with between 11 and 17 pg/mL, and the increases were modest, less than 5 pg/mL.

Similar to sNfL, pre‐IAHSCT levels of sGFAP were elevated compared with controls (Geomean 107.4 vs. 50.7 pg/mL, Unpaired t test *P* = 0.0001, Fig. 1B). At 3 months, sGFAP was increased relative to baseline in 13/15 patients (geomean 107.4 pg/mL, *P* = 0.0004, 95% CI for the ln‐transformed mean difference 0.2597 to 0.8574). The baseline to 3‐month increase in geometric mean NfL from 107.4 to 187.7 pg/mL represented a 74.8% increase. As with sNfL, there were two patients with a baseline to 3‐month decrease in sGFAP levels; these were the same samples where sNfL levels had decreased. Unlike sNfL, 6‐month sGFAP remained elevated relative to baseline in 17/19 patients (Geomean 150.0 pg/mL, *P* = 0.0014, CI 0.12 to 0.54). At 12 months post‐IAHSCT, although GFAP levels had reduced from their 3‐month peak (Geomean 134.6 pg/mL, *P* = 0.0173, CI −0.6639 to −0.06067) they remained higher than baseline levels in 18/22 patients (although this difference was not significant), and elevated relative to controls (Unpaired T test *P* = 0.0001).

### Correlates of sNfL changes

We looked for correlations between early serum biomarker changes with clinical and MRI markers of possible neurotoxicity. Although the small sample size did not permit a multivariate regression analysis, we performed several nonparametric (Spearman) correlations of the baseline to 3‐month difference in sNfL (hereafter, 3‐month ΔsNfL, Table 2). Three‐month ΔsNfL correlated with changes in sGFAP at the same time points (*r* = 0.071, *P* = 0.00018). Furthermore, 3‐month ΔsNfL correlated inversely with baseline sNfL levels; the greatest increases in sNfL were observed in patients with the lowest baseline levels and vice versa (*r*=−0.78, *P* = 0.0006). Additionally, 3‐month ΔsNfL correlated with the total dose of busulfan administered during transplant conditioning (*P* = 0.034), EDSS worsening in the first 6 months (*P* = 0.041), and the increase in whole brain volume loss at 6 months (*P* = 0.044), particularly due to grey matter loss (*P* = 0.0023). Further analysis of the correlation between 3‐month ΔsNfL and grey matter volume loss post‐IAHSCT revealed correlations at all early time points (months 1 (*P* = 0.0161), 2 (*P* = 0.017), 4 (*P* = 0.0045), and 6 (*P* = 0.0023)), but not 9 or 12 months post‐IAHSCT, highlighting that associated increases in both 3‐month ΔsNfL and MRI grey matter volume loss are temporary phenomena in the first year following IHASCT.

**Table 2:** Spearman correlation between 3‐month ΔsNfL (3‐month – baseline level) and other serological, MRI, and clinical markers of possible neurotoxicity

	Correlation coefficient (95% CI)	*P* value
Change in GFAP (BL‐3m)	0.73 (0.43 to 0.89)	0.0018
Baseline NfL level	−0.78 (−0.80 to −0.13)	0.006
MRI Grey matter volume loss 6 months post‐IAHSCT	0.74 (0.43 to 0.89)	0.0023
Total busulfan dose	0.56 (0.14 to 0.81)	0.034
EDSS score change 6 months post‐IAHSCT	0.52 (0.086 to 0.79)	0.041
MRI whole brain volume loss 6 months post‐IAHSCT	0.58 (0.13 to 0.87)	0.045
Symbol Digit Modalities Test score change 6 months post‐IAHSCT	−0.73	0.17 (ns)
Computerized Test of Information Processing score change 6 months post‐IAHSCT	0.89	0.11 (ns)
Paced Auditory Serial Addition Test score change 6 months post‐IAHSCT	−0.36	0.15 (ns)

Although none of the comparisons of 3‐month Δ sNfL and cognitive scores reached statistical significance, baseline to 6‐month RCI scores for all three tests of cognitive processing speed that we used showed correlations in the anticipated direction (Table 2). For the Symbol Digit Modalities Test (SDMT) and Paced Auditory Serial Addition Test (PASAT), where higher scores represent a better result, we found non‐significant negative correlations (SDMT, *r* = −0.73, *P* = 0.17; PASAT *r* = −0.36; *P* = 0.15). For the Computerized Test of Information Processing (CTIP), where higher scores represent worse functioning, we found a nonsignificant positive correlation (*r* = 0.89, *P* = 0.11). These findings, may suggest that with more patients we may find that rising NfL levels indeed correlate with cognitive processing post‐IAHSCT. Otherwise, we did not find correlations with raw test scores and RCI scores for the Brief Visuospatial Memory Test–Revised, Hopkins Verbal Learning Test–Revised, Judgment of Line of Orientation, Delis‐Kaplan Sorting Test, and letter fluency and semantic fluency by animal naming. Similarly, we found no correlation with 3‐month change in sNFL with quantitative fatigue (Fatigue Impact Score) and quality of life (MS‐QOL‐54, physical and mental composites) at the same time points. There was no correlation between 3‐month Δ and sNfL demographic variables including age and MS subtype (relapsing remitting vs. secondary progressive).

**Figure 2:**
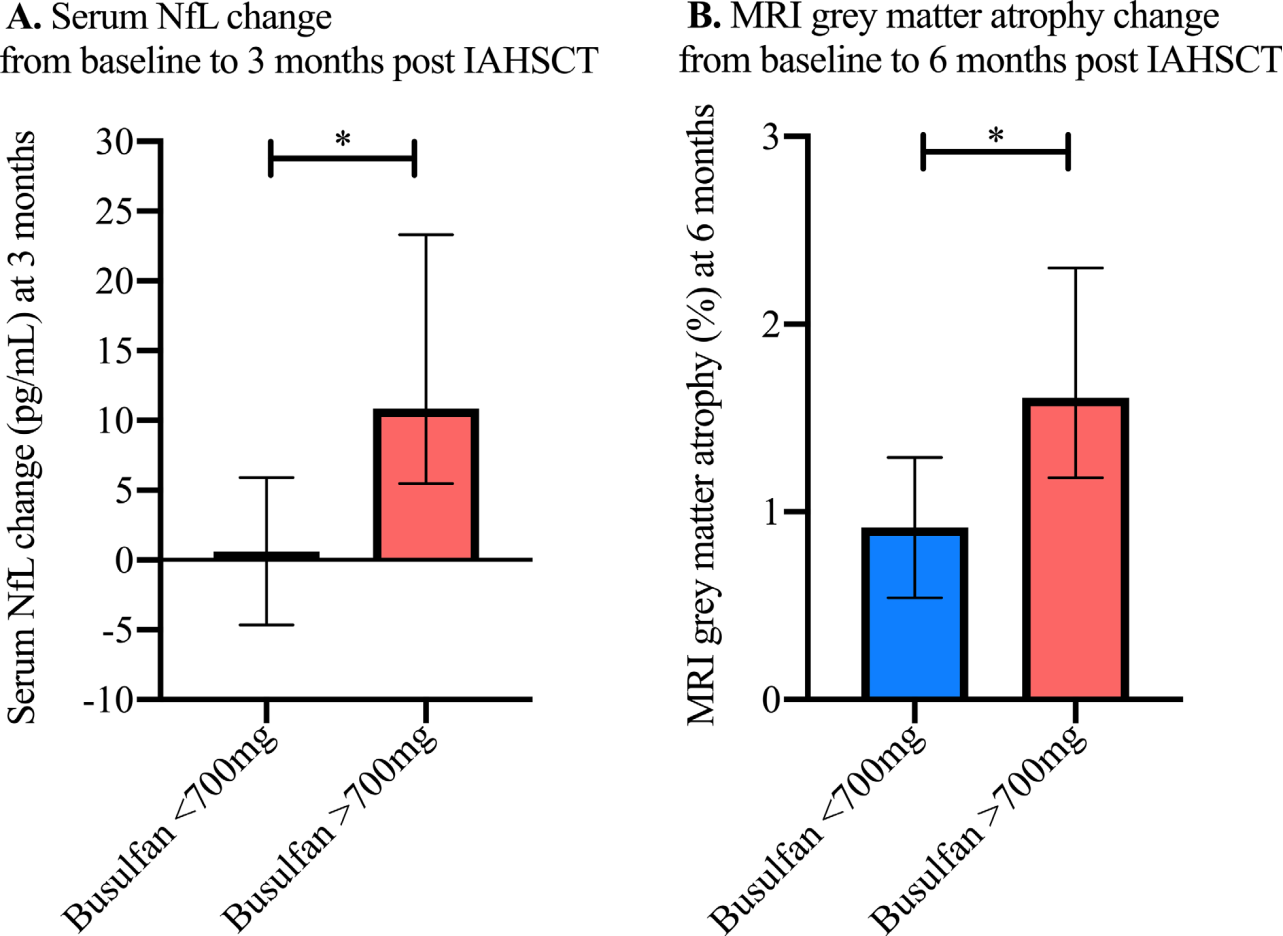
Doses of busulfan higher than the median of 700 pg/mL were found to be associated with a greater increase from baseline to 3‐month serum NfL levels (A, Mann–Whitney *P* = 0.037) and 6‐month percentage grey matter atrophy (B, *P* = 0.019). Error bars = Standard error of the mean. * signifies a *P*‐value ≤ 0.05.

Patients received a wide range of total busulfan doses based on body weight due to a change in the protocol after the first 10 patients enrolled (median 705 mg, minimum 464 mg, and maximum 1176 mg). To enable binary categorical comparison of median busulfan dose, we predefined patients as having received ‘low’ (<700 mg, *n* = 11) or ‘high’ (>700 mg, *n* = 11) doses. Using these groups and nonparametric categorical analyses we compared the 3‐month ΔsNfL (Fig. 2A) and baseline to 6‐month differences in grey matter volume loss (Fig. 2B). Patients who had received> 700mg of busulfan had a larger 3‐month ΔsNfL (*P* = 0.037) and greater extent of grey matter volume loss (*P* = 0.019).

**Figure 3:**
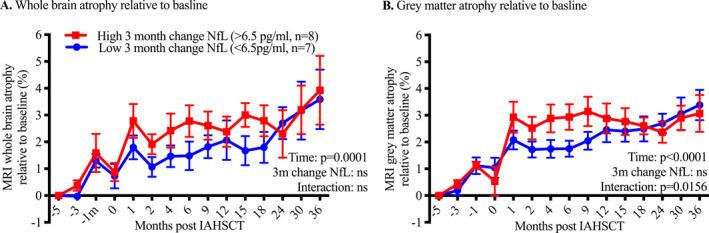
High (>6.5 pg/mL) compared with low (<6.5 pg/mL) change in serum NfL from baseline to 3 months was not associated with differential rates of whole brain atrophy over time (A). The same was true for white matter atrophy (data not shown). However, looking in the grey matter, the interaction was significant (B). In the first year post‐IAHSCT, patients with a high 3‐month change in NfL experienced more grey matter atrophy. The curves had reconverged by 12 months and then followed a similar trajectory. Error bars = Standard error of the mean.

Using the median 3‐month ΔsNfL (+6.5 pg/mL) to divide the cohort, patients who experienced a 3‐month ΔsNfL of >6.5 pg/mL were considered to have a ‘high’ 3‐month NfL change, whereas those with a 3‐month ΔsNfL of <6.5 pg/mL or a decrease in levels were considered to have a ‘low’ change. Using a mixed‐effects model (random effect for subjects and fixed effects for other terms) we compared rates of whole brain, white and grey matter volume loss over time (Fig. 3). While there was a significant increase in % volume loss over time in both whole brain white and grey matter (*P* < 0.0001, white matter volume loss not shown), the interaction of time – 3‐month ΔsNfL – was only significant for grey matter volume loss (*P* = 0.0156); patients with 3‐month NfL increases >6.5 pg/mL experienced increased rates of grey matter volume loss over the first year following IAHSCT. Moreover, the two curves diverged at 1‐month and reconverged by 12 months, perhaps indicating a temporary early insult causing the divergence in grey matter atrophy.

## Discussion

IAHSCT remains a very intensive therapy for a select minority of MS patients with aggressive disease. Here, we show that in the first few months following IAHSCT for aggressive MS, there are transient increases in serum markers of both neuroaxonal and glial cell injury (sNfL and sGFAP). Moreover, the increases in sNfL correlated with the total busulfan dose, transient worsening of post‐treatment EDSS score as well as MRI volume loss, especially in the grey matter.

Although others have independently observed rises in brain atrophy and serum markers of neurotoxicity following other hematopoietic stem cell transplantation regimens for MS,^24^ this study is the first to correlate these increases with doses of chemotherapy administered. There was a moderate positive linear relationship between NfL rise and EDSS score worsening, and three independent cognitive tests of information processing speed (PASAT, SDMT, and CTIP) showed trends toward worse scores that did not reach significance. This perhaps gives credence to the ‘chemo fog’ many patients transiently describe post‐IAHSCT. Our study was limited by small sample size, which prohibited a multivariate comparison to deeper probe the relationships among chemotherapy, blood, and MRI markers of neurodegeneration and clinical outcomes.

The 32% increase in median sNfL we measured from baseline to 3 months seems much more modest than the 79% increases in the related serum neurofilament heavy (sNfH) chain found 1‐month posttreatment in the study by Petzold et al.^10^ sNfH levels in their study remained persistently elevated beyond 12 months, while in our study sNfL levels had reduced to lower than baseline levels by 12 months. Comparison between the studies is limited in part given the different measurements of NfH and NfL. Moreover, a different conditioning regimen was utilized including whole body irradiation in the Petzold study, which may well have compounded, as well as extended the effect of temporary exposure to a chemotherapeutic agent. Whole body irradiation is no longer often used for MS due to side effects and relatively poorer suppression of inflammatory activity.

The single greatest weakness of our study is the lack of a control group of patients undergoing IAHSCT for non‐MS reasons, such as non‐MS autoimmune conditions (e.g., myasthenia gravis or neuromyelitis optica) or hematological malignancy. Although no such cohort was available for retrospective analysis, this could have aided the interpretation of our study. Both sGFAP and sNfL are nonspecific markers and can reflect many types of underlying brain insult. In this study, baseline NfL levels were already elevated compared with controls as a marker of intense disease activity in these patients which warranted IAHSCT.^25^ These patients had no detectable evidence of MS disease‐related inflammation post‐IAHSCT, therefore it is unlikely that disease contributed to the early increases in these biomarkers. It is also plausible that the already damaged CNS in patients with aggressive MS may be especially vulnerable to the toxic effects of chemotherapy. Pointing more toward a nonspecific effect of the conditioning drugs, our group has previously reported that a patient with CNS‐sparing lymphoma undergoing a near identical IAHSCT regimen had an annualized rate of atrophy of 6.0%/year, which was in the higher end of the range of atrophy measurements seen in nine MS patients during a similar treatment interval (median 3.2%/year).^5^ Suggestive of an enhanced vulnerability of the MS patients, in the radio‐ablative study reported by Petzold et al.^24^ which did include a non‐MS IAHSCT control group, while increases in sNfH were seen in both MS patients and non‐MS patients undergoing IAHSCT, the greatest increases were seen in the MS patients.

The 3‐month peak in sGFAP levels post‐IAHSCT is very likely a manifestation of CNS toxicity similar to the increased sNfL levels, rising in the first 3 months, then gradually decreasing. However, by 12 months, levels were near to pretreatment levels but still much higher than controls, a curious finding. While the dynamics and significance of serum levels of GFAP is not well understood, GFAP is considered the archetypal marker of reactive astrocytes,^26^, ^27^ which are now understood to encompass spectrum of phenotypes both deleterious and beneficial in MS.^28^ One possibility is that the sustained levels of GFAP at 12 months could represent a failure of IAHSCT to control ongoing astroglial damage, either related to MS activity^17^, ^29^ or the treatment toxicity. However, this hypothesis does not fit with the clinical picture, as by 12 months, the majority of these patients remained clinically stable if not improved slightly from pre‐IHASCT disability, with no new MRI lesions and a normalization of brain atrophy rates similar to that of healthy controls.^11^, ^30^ Although not mutually exclusive, another possibility is the ongoing elevation in GFAP post‐IAHSCT could represent more benign or even beneficial role of astrocytes in these patients. Unlike neurons, GFAP‐expressing reactive astrocytes are known to be a dynamic cell population^31^ which persist for many years even in chronically inactive MS lesions.^32^ Further confounding the situation, CD‐34‐positive autologous hematopoietic stem cells used as part of the IAHSCT itself are capable of differentiation into GFAP‐expressing reactive astrocytes.^33^ We postulate that while peripheral levels of NfL represent the loss of finite pool of neuronal axons, levels of GFAP more likely reflect the turnover of reactive astrocytes carrying out a spectrum of roles in MS lesions.

Due to a lack of comparative prospective studies, consensus of how to minimize IAHSCT morbidity and mortality against achieving the desired treatment effect is yet to be reached. This study is the first published attempt to directly quantify putative markers of toxicity in one of these cohorts, data which may help inform this debate. The main differences between protocols relate to the conditioning agents and their doses, which correspondingly impact both the degree of immunoablation and myeloablation as well as toxicity.^1^ While the Canadian protocol involved busulfan, high doses of cyclophosphamide and antithymocyte globulin (ATG) for conditioning, other regimens use a less toxic regimen of lower‐dose cyclophosphamide and ATG, and no busulfan whatsoever.^34^, ^35^ Although the toxicity profile of busulfan for IAHSCT in MS^3^ has been optimized through improved patient selection, lower dosages, and IV administration, this alkylating agent is known to cause neurotoxicity at higher doses.^36^ While lower intensity regimens that do not contain busulfan still attain much higher rates of 2‐year ‘No Evidence of Disease Activity” compared with conventional pharmacotherapies,^2^ in the most recent study reported by Burt et al., 4/53 and 8/53 patients receiving the nonmyeloablative IAHCT regimen experienced a clinical relapse by 2 years and 4 years, respectively. This compares to the higher‐intensity Canadian group where not a single patient included in the original study^11^ experienced a relapse or new MRI lesion after nearly 20 years of follow‐up, indicating a more durable treatment response.

Although we see transient neurotoxicity likely related to busulfan in this study, this has to put in the context of their highly active refractory disease. While the long‐term effects of chemotherapeutic neurotoxicity are not known, in cases of aggressive MS, short‐term chemotherapy‐related neuronal toxicity may be a risk that is greatly offset by the benefit of long‐term freedom from disease‐related inflammation.

## Author Contributions

ST was involved in conception and design of the study, acquisition and analysis of data, and drafting manuscript and figures. HL, SN, and DA were involved in acquisition and analysis of MRI data. GB, MA, MB, CAR, and HA were involved in acquisition and analysis of clinical data. JB and LW were involved in acquisition and analysis of cognitive data. DT, RB, and VT‐C were involved in acquisition and analysis of sNfL and GFAP data. AB‐O was involved in study conception and design. MSF was involved in conception and design of the study as well as drafting the manuscript.

## Conflict of Interest

There were no relevant conflicts of interest related to this study.
